# A Novel Severity Score Index for Febrile Neutropenic Patients with Colorectal Diseases

**DOI:** 10.1155/2019/4175960

**Published:** 2019-03-24

**Authors:** Camila Perazzoli, Rogério S. Parra, Marley R. Feitosa, Enrico Sfoggia, Belinda Pinto Simões, José J. R. Rocha, Omar Féres

**Affiliations:** ^1^Department of Surgery and Anatomy, Ribeirão Preto Medical School, University of São Paulo, Brazil; ^2^Department of Internal Medicine, School of Medicine of Ribeirão Preto, University of São Paulo, Ribeirão Preto, SP, Brazil

## Abstract

**Introduction:**

Abdominal and anorectal disorders may be the cause of clinical decompensation in neutropenic febrile patients, particularly those with hematologic diseases. Infection is a cause for concern for the colorectal surgeon. Some conditions have few manifestations and can lead to death within a short period of time. This study presents the novel colorectal disorder severity score for febrile neutropenic patients.

**Materials and Methods:**

This was a case series study analyzing the medical records of 897 patients admitted to the Hematology and Hematopoietic Stem Cell Transplant Unit in a university hospital between the years 2008 and 2013. Seventy-four episodes of febrile neutropenia in 69 patients diagnosed with an abdominal or anorectal infection site were eligible for the study. The new scoring system proposed here is based on the author's clinical experience and an extensive literature review. In addition to the extensive literature review, effect measures were calculated, and a statistical analysis was performed. Based on an evaluation of common biological plausibility criteria, five factors were selected as the main predictors of hospital mortality in febrile neutropenic patients with colorectal disease.

**Results:**

The proposed score demonstrated increased mortality as the condition worsened as reflected by an increasing score (Fisher's exact test: 0.001). When considering the logistic model for the probability of death by score level, the AUC value was 0.82 (0.72-0.925), and the Hosmer-Lemeshow statistic value was 2.3, *p* = 0.806.

**Conclusion:**

The proposed scoring system allows prediction of the likelihood of death during hospitalization for febrile neutropenic patients with an abdominal and anorectal focus. New studies on the subject are required, and the proposed scoring scale must be validated on a larger and different sample of patients.

## 1. Introduction

The diagnosis and management of a surgical abdomen and anal diseases in patients with febrile neutropenia are quite difficult because of complications and treatment of the underlying disease. Severe and prolonged neutropenia is more common in patients who are undergoing both acute leukemia induction chemotherapy and conditioning or pregrafting for hematopoietic stem cell transplantation (HSCT) [1, 2].

The most popular tool used to predict the risk of complications associated with a febrile neutropenia episode is the Multinational Association of Supportive Care in Cancer (MASCC) score; individuals with a *score* ≥ 21 points are considered low risk and can receive empirical antibiotic therapy at home. This approach reduces treatment costs and improves patients' quality of life (Table 1) [3]. According to the MASCC score and the numerous references related to the topic in the literature, patients with malignant hematologic diseases (i.e., nonsolid tumors) are at the greatest risk of developing febrile neutropenia since, in addition to receiving treatment with cytotoxic agents, myelopoiesis is damaged by changes caused by the underlying disease. Furthermore, chemotherapy regimens for solid tumors generally cause shorter-lasting neutropenia than chemotherapy regimens for hematologic malignancies. An estimated 10% to 50% of solid tumors and more than 80% of hematologic malignancies will involve at least one episode of febrile neutropenia during a course of chemotherapy [1].

Neutropenic enterocolitis (NE) affects the terminal ileum and/or colon (cecum) and is almost exclusively found in neutropenic patients [4]. Enterocolitis is the most common abdominal disease in patients with febrile neutropenia and affects up to 50% of patients after chemotherapy [5]. The occurrence of NE involves a combination of factors, including mucosal injury, cytotoxic drugs, severe neutropenia, and the defense system of the affected patient. The main symptoms are fever, diarrhea, and abdominal pain (usually in the lower right quadrant) [6], and less frequently, abdominal distension, nausea, vomiting, melena, and rectal bleeding are observed [7, 8]. Signs of peritoneal irritation or shock suggest the possibility of bowel wall perforation [9, 10].

The diagnosis of NE is based on clinical history combined with an abdominal CT scan with contrast [11]. The main tomographic findings are as follows: colon lumen distension, swelling of the cecum wall, mesocolonic or mesenteric infiltration of the terminal ileum, intestinal pneumatosis, localized perforation, free perforation in the cavity, and abscess. CT can rule out other disorders, such as acute appendicitis, periappendicular abscess, and pseudomembranous colitis [9, 11]. In the absence of complications, NE treatment includes a nasogastric tube, hydration, and broad-spectrum antibiotics [12]. In the presence of peritonitis, free perforation, persistent gastrointestinal bleeding, or clinical deterioration, a surgical approach is indicated [5, 13, 14].

Anorectal disorders are distinguished as either septic (fistulas, abscesses, and Fournier's syndrome) or nonseptic (fissures and hemorrhoidal disease) due to the different clinical outcomes and mortality between the two types. Septic perianal disease in neutropenic patients often requires surgery and is associated with a mortality rate of up to 78% [15]. Septic perianal conditions present and progress differently in neutropenic patients. Anorectal abscesses typically develop accompanied only by local pain and fever. Rectal or any other more invasive type of examination must be performed with caution due to a risk of encouraging bacterial translocation and worsening sepsis [16–18].

The frustrating symptomology combined with the belief that sepsis may develop secondarily to diagnostic and therapeutic procedures can lead to death within a short amount of time for immunocompromised patients [12]. The mortality of patients with acute leukemia and anorectal disease varies from 45-78%, and this condition must be treated in-hospital [17, 19]. The literature on the subject is unclear in terms of which diseases benefit from surgical management and the optimal time to pursue this course of action [12, 20]. A correlation exists between the number of circulating granulocytes and the incidence of anorectal infection. The incidence of perianal infections is 7.3% in patients with acute leukemia, compared to 0.92% in patients with chronic leukemia [18].

Anorectal diseases and neutropenic enterocolitis can be fatal in febrile neutropenic patients. Colorectal surgeons are frequently asked by hematologists to evaluate febrile neutropenic patients with an abdominal or anorectal focus. The indistinct symptomology, combined with the limited ability to perform physical examinations, the severe and unsatisfactory prognosis, and the lack of references in the medical literature, indicates the need for studies on this subject. No severity and prognostic scoring systems are available for patients with febrile neutropenia and colorectal diseases. The aim of this study was to create a severity score for abdominal and anorectal diseases in febrile neutropenic patients.

## 2. Method

This was a case series study based on a review of the medical records of 897 patients hospitalized with febrile neutropenia in a tertiary hospital between February 2008 and February 2013. Seventy-four episodes of febrile neutropenia in 69 patients diagnosed with an abdominal or anorectal infection site were eligible for the study. Based on the evaluation of the study population, an MS Excel spreadsheet was created that considered quantitative variables (age, neutropenia duration, and severity of neutropenia) and qualitative variables (gender, diagnosis of abdominal or anorectal disease, treatment of colorectal condition, baseline hematologic diagnosis, hematologic disease treatment phase, blood culture, comorbidities, and in-hospital mortality during the study period).

The collected data were pooled and subjected to calculations of absolute (AR) and relative (RR) risks of in-hospital mortality. The objective was to evaluate the sample characteristics related to the in-hospital death outcome and to calculate the statistical significance of these associations. Five factors were selected as major predictors of in-hospital mortality based on the literature on the subject, common biological plausibility criteria, the author clinical experience, the calculations of effect measures, and statistical analysis of the sample data (Table 2(a)). The five selected factors were then stratified with sequential and increasing numerical values. The scores attributed to each studied case were added, and the final result was divided into risk groups. The groups were compared to each other by analyzing the AR, RR, and chi-square/Fisher exact test results. *p* values ≤ 0.05 were accepted as statistically significant. Lastly, the factors selected by the authors were tested for their discriminative power using logistic regression, area under the receiver operating characteristic (ROC) curves, and the Hosmer-Lemeshow (HL) statistic.

## 3. Results

The studied population was divided into three groups according to points assigned on the proposed severity scale (Table 2(b)). Group I, whose total score was between 5 and 8 points, included 10 cases, and no deaths were recorded in this group during the hospitalization period under study. Group II, whose total score ranged from 9 to 12 points, included 32 cases. Two deaths were recorded in this group during the hospitalization period under study. The AR of mortality in group II was 6.2%. Group III, whose total score was between 13 and 16 points, included 32 cases, and 13 patients died during the studied hospitalization period. The AR of mortality in group III was 40%.

**Figure 1:**
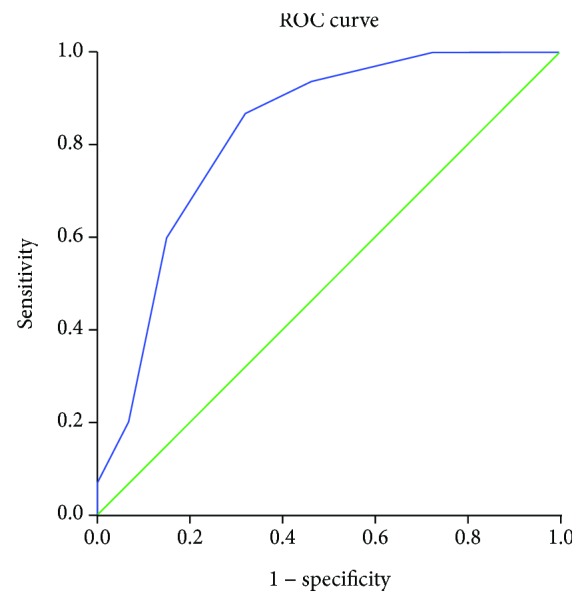
Receiver operating characteristic (ROC) curve to predict the risk of mortality according to group index.

Calculating the overall test results, the Fisher exact test value was 0.001. By analyzing the groups, mortality was found to increase as the score increased. When considering the logistic model for the probability of death by score level, the AUC value was 0.82 (0.72-0.925), and the HL statistic value was 2.3, *p* = 0.806 (Figure 1).

## 4. Discussion

This is the first study to propose a severity score for colorectal disease in patients with febrile neutropenia. Neutropenic enterocolitis and anorectal diseases, such as abscesses and fistulas, are well-known gastrointestinal and colorectal infectious complications during the treatment of hematologic malignancies. An increasing score reflected increasing mortality. Clinical/laboratory scores are important in daily practice for objective decision-making and prognostic evaluation.

In clinical practice, especially in intensive care units (ICUs), many score scales have been developed and refined to quantify disease severity, evaluate prognosis, direct therapeutic interventions, and coordinate the distribution of resources. The Acute Physiology and Chronic Health Evaluation (APACHE) system, for example, uses physiological data, previous morbid conditions, and information regarding the nature of the current disease to predict the probability of in-hospital death of a critically ill patient [21]. Based on the APACHE score, we propose a prognostic score that is aimed at predicting the risk of in-hospital death of febrile neutropenic patients with a diagnosed abdominal or anorectal disorder. As with the APACHE score, we selected in-hospital death as the dependent variable because it is easy to assess, is dichotomous (died or survived), and is the primary outcome of interest. The independent variables related to the dependent variable and used to constitute the score (duration of neutropenia, diagnosis of the underlying disease, number of neutrophils, current therapeutic modality, and colorectal infection focus) were selected based on the literature on the subject, the authors' clinical experience, sample analysis, and calculation and analysis of the relative risk of in-hospital mortality.

As with the APACHE score and its refinements, the data collected and selected to develop this score were subjected to an evaluation of discrimination and calibration, which are statistical tools that demonstrate the behavior and reliability of the results. For the APACHE IV score, the discrimination, or area under the ROC curve, is 0.88, and calibration using the HL statistic has a value of 16.9, with *p* = 0.08 [21]. In terms of the proposed score, considering the logistic model of the probability of death by score level, the ROC curve value was 0.82 (0.72 to 0.93), and the HL statistic was 2.3, *p* > 0.806. ROC curve values above 0.8 suggest a good prognostic score, and *p* values > 0.05 for the HL statistic show that the score is reliable. Moreover, when the score was subjected to statistical analysis, mortality increased as the score increased (Fisher's exact test, 0.001). Patients with neutropenia are unable to produce a significant inflammatory response, and serious infections consequently occur, with few signs and symptoms. In these patients, fever is often the only sign of infection, and the clinical outcome can worsen quickly and progressively, progressing to sepsis, septic shock, and death [1, 2, 22]. The critical point for improvement of these outcomes is to recognize the condition of febrile neutropenia as soon as possible, collect laboratory tests and cultures, and start broad spectrum antibiotics immediately [1, 9]. The conditions that affect adequate myelopoiesis predispose patients to a persistent inflammation of the mucosa, which is called mucositis. This manifestation increases a patient's vulnerability to invasive infections caused by bacterial translocation, usually enterobacteria, through the weakened surface of the intestinal mucosa [23]. This combination of factors facilitates the emergence of potentially very serious colorectal infectious foci, such as neutropenic enterocolitis and anorectal diseases [5, 23, 24].

A pertinent observation in relation to the design of this study is the fact that it refers to cases and not to patients. This position is in accordance with the literature on the subject because the characteristics of each febrile neutropenia episode studied have little or no effect on the occurrence of new episodes once the marrow is rehabilitated (which occurs between 7 and 10 days after chemotherapy or 15 and 45 days after allogeneic HSCT). Since we evaluated only in-hospital mortality, each febrile neutropenia episode with abdominal or anorectal disease was evaluated separately.

When considering the variables selected for the score, we identified a greater weighting of events related to the underlying disease compared to coloproctological characteristics. This can be explained by the severity of the studied blood disorders and the fact that the hematology service at this university hospital is a national tertiary reference center in the field, which ultimately receives severe cases and those refractory to less aggressive therapies.

Another important consideration is related to characteristics such as length of hospital stay, bacterial growth in the blood culture, and growth of Gram-negative bacteria in the blood culture. These characteristics appear to be related to adverse outcomes in febrile neutropenic patients according to the literature, but they were not selected to form part of the present severity score because we found that specifying the hospitalization time specifically associated with the febrile neutropenia episode under study was difficult (particularly in cases of long hospitalizations in which one infectious condition followed another) and because we did not identify blood culture collection in all episodes of febrile neutropenia from the time of the initial manifestations of the infectious focus [1, 25–27].

Hematologic evaluations are routinely performed in hospitalized febrile neutropenic patients with suspected or confirmed colorectal disease as the cause of their clinical worsening. Some patients exhibit rapid clinical deterioration, and mortality rates are high. The medical literature on the subject is scarce. The new severity score was predictive of mortality in this group of patients.

This study has some limitations. First, the study was not initially conceived to develop a mortality score but to conduct a case series, analyze the sample characteristics, and compare the results with previously published data. However, as we collected data and reviewed studies on the subject, we found that among the literature with a specific focus on colorectal diseases, the number of cases studied was limited, and the available data contained no univariate or multivariate analysis of the sample characteristics. Therefore, we abandoned the initial logistic regression model and proceeded with validation (discrimination and calibration) of the data with a pilot study design. Second, we included patients with oncohematologic diseases, benign hematologic diseases, and autoimmune diseases in the development of the score. In particular, for autoimmune diseases, numerous theses, publications, consensus papers, and meetings show that treatment of serious and refractory autoimmune diseases can be carried out successfully using HSCT, similar to other related pathologies. Although we included three different disease groups at the time of evaluation, all patients were immunosuppressed (most often severely).

In conclusion, the proposed scoring system allows prediction of the likelihood of in-hospital death for febrile neutropenic patients with an identified abdominal and anorectal focus. New studies on the subject are required, and the proposed scoring system must be validated on a larger and different sample of patients.

## Figures and Tables

**Table 1:** MASCC risk index factors and weights [3].

Characteristic	Weight
Burden of febrile neutropenia with no or mild symptoms^I^	5
No hypotension (systolic BP > 90 mmHg)	5
No chronic obstructive pulmonary disease^II^	4
Solid tumor or hematological malignancy with no previous fungal infection^III^	4
No dehydration requiring parenteral fluids	3
Burden of febrile neutropenia with moderate symptoms^IV^	3
Outpatient status	3
Age < 60 years	2

MASCC (Multinational Association of Supportive Care in Cancer). ^I^Burden of febrile neutropenia refers to general clinical status as influenced by the febrile neutropenic episode. It is evaluated in accordance with the following scale: no symptoms (5), mild symptoms (5), moderate symptoms (3), severe symptoms (0), and moribund (0). ^II^Chronic obstructive pulmonary disease means active chronic bronchitis, emphysema, decrease in FEVs, need for oxygen therapy, and/or steroids and/or bronchodilators. ^III^Previous fungal infection means demonstrated fungal infection or empirically treated suspected fungal infection. ^IV^The points attributed to the variable “burden of febrile neutropenia” are not cumulative. Thus, the maximum theoretical score is therefore 26. A score of ≥21 is considered low risk and a score of <21 as high risk (positive predictive value of 91%, specificity of 68%, and sensitivity of 71%).

**Table 2:** (a) Five main predictors of in-hospital mortality according to the medical literature, common biological plausibility criteria, clinical experience of the authors, calculations of effect measures, and statistical analysis of the sample data. (b) Three groups according to points assigned on the proposed severity scale. Group I (5-8 points), group II (9-12 points), and group III (13-16 points).

(a) Variable		Score
Underlying disease	MM, ES, EM	1
	NHL (moderate and low grade), HL, CLL	2
	AA, MDS, accelerated phase CML	3
	AML, ALL, high-grade NHL, blastic phase CML	4
Neutropenia classification (number of neutrophils)	Mild: between 1000 and 501 cells/mm^3^	1
	Moderate: between 500 and 100 cells/mm^3^	2
	Severe: ≤100 cells/mm^3^	3
Duration of neutropenia	≤7 days	1
	8-14 days	2
	≥15 days	3
Current therapeutic modality	Autologous HSCT/other treatments	1
	Combination drug chemotherapy	2
	Allogenic HSCT	3
Colorectal disorder	Nonseptic anorectal focus	1
	Septic anorectal focus	2
	Abdominal focus	3

(b) Group	Score	
I	5-8	
II	9-12	
III	13-16	

Based on this process, a severity score scale was created to estimate the risk of in-hospital death of febrile neutropenic hematologic patients with a diagnosed abdominal or anorectal focus.

## Data Availability

Data were collected by the computerized hospital system and medical records. All data used and analyzed during the current study can be send, if necessary, as e-mail request (corresponding author). All data analyzed during this study are included in this published article.
